# 2-Methyl­phenyl 4-methyl­benzoate

**DOI:** 10.1107/S1600536808022733

**Published:** 2008-07-23

**Authors:** B. Thimme Gowda, Sabine Foro, K. S. Babitha, Hartmut Fuess

**Affiliations:** a Department of Chemistry, Mangalore University, Mangalagangotri 574 199, Mangalore, India; b Institute of Materials Science, Darmstadt University of Technology, Petersenstrasse 23, D-64287 Darmstadt, Germany

## Abstract

The conformation of the C=O bond in the title compound 2MP4MBA, C_15_H_14_O_2_, is *anti* to the *ortho*-methyl group in the phen­oxy ring. The bond parameters in 2MP4MBA are similar to those in 3-methyl­phenyl 4-methyl­benzoate (3MP4MBA), 4-methyl­phenyl 4-methyl­benzoate (4MP4MBA) and other aryl benzoates. The dihedral angle between the two aromatic rings in 2MP4MBA is 73.04 (8)°.

## Related literature

For related literature, see Gowda *et al.* (2007 ▶, 2008 ▶); Nayak & Gowda (2008 ▶).
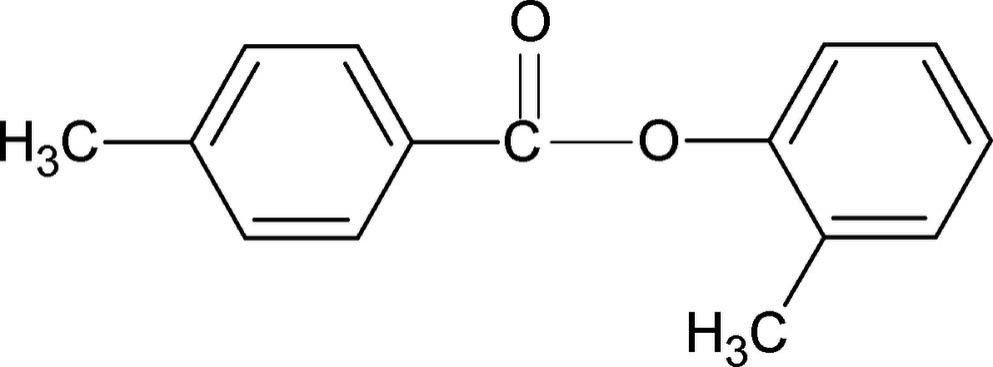


## Experimental

### 

#### Crystal data

C_15_H_14_O_2_
*M* *_r_* = 226.26
Monoclinic, 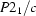

*a* = 11.690 (2) Å
*b* = 9.670 (1) Å
*c* = 11.478 (2) Å
β = 104.50 (2)°
*V* = 1256.2 (3) Å^3^
*Z* = 4
Mo *K*α radiation
μ = 0.08 mm^−1^
*T* = 299 (2) K
0.50 × 0.46 × 0.20 mm

#### Data collection

Oxford Diffraction Xcalibur diffractometer with a Sapphire CCD detector
Absorption correction: multi-scan (*CrysAlis RED*; Oxford Diffraction, 2007 ▶) *T*_min_ = 0.968, *T*_max_ = 0.989
7861 measured reflections
2529 independent reflections
1385 reflections with *I* > 2σ(*I*)
*R*_int_ = 0.021

#### Refinement

*R*[*F* ^2^ > 2σ(*F* ^2^)] = 0.053
*wR*(*F* ^2^) = 0.200
*S* = 1.04
2529 reflections
181 parameters
H atoms treated by a mixture of independent and constrained refinement
Δρ_max_ = 0.21 e Å^−3^
Δρ_min_ = −0.15 e Å^−3^

### 

Data collection: *CrysAlis CCD* (Oxford Diffraction, 2007 ▶); cell refinement: *CrysAlis RED* (Oxford Diffraction, 2007 ▶); data reduction: *CrysAlis RED*; program(s) used to solve structure: *SHELXS97* (Sheldrick, 2008 ▶); program(s) used to refine structure: *SHELXL97* (Sheldrick, 2008 ▶); molecular graphics: *PLATON* (Spek, 2003 ▶); software used to prepare material for publication: *SHELXL97*.

## Supplementary Material

Crystal structure: contains datablocks I, global. DOI: 10.1107/S1600536808022733/bx2161sup1.cif

Structure factors: contains datablocks I. DOI: 10.1107/S1600536808022733/bx2161Isup2.hkl

Additional supplementary materials:  crystallographic information; 3D view; checkCIF report

## Figures and Tables

**Fig. 1.**
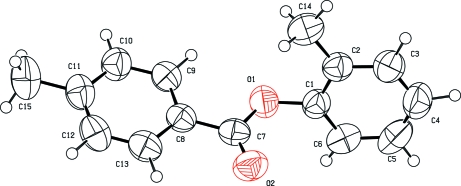
Molecular structure of the title compound, showing the atom labeling scheme. The displacement ellipsoids are drawn at the 50% probability level. The H atoms are represented as small spheres of arbitrary radii.

**Fig. 2.**
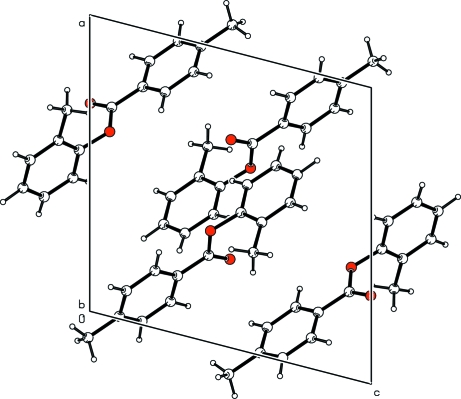
Molecular packing of the title compound.

## supplementary crystallographic information

### Comment

In the present work, as part of a study of the substituent effects on the crystal structures of aryl benzoates (Gowda *et al.*, 2007; 2008), the structure of 2-methylphenyl 4-methylbenzoate (2MP4MBA) has been determined. The conformation of the C=O bond in 2MP4MBA is *anti* to the *ortho*-methyl group in the phenolic benzene ring (Fig. 1). The bond parameters in 2MP4MBA are similar to those in 3-methylphenyl 4-methylbenzoate (3MP4MBA), 4-methylphenyl 4-methylbenzoate (4MP4MBA) (Gowda *et al.*, 2007) and other aryl benzoates (Gowda *et al.*, 2008). The dihedral angle between the benzene and benzoyl rings in 2MP4MBA is 73.04 (8)°, compared to the values of 56.82 (7)° in 3MP4MBA and 63.57 (5)° in 4MP4MBA. The packing diagram of molecules in the crystal structure is shown in Fig. 2.

### Experimental

The title compound was prepared according to a literature method (Nayak & Gowda, 2008). The purity of the compound was checked by determining its melting point. It was characterized by recording its infrared and NMR spectra (Nayak & Gowda, 2008). Single crystals of the title compound used for X-ray diffraction studies were obtained by slow evaporation of an ethanolic solution at room temperature.

### Refinement

The H atoms of the methyl groups were positioned with idealized geometry using a riding model with C—H = 0.96 Å. The other H atoms were located in difference map, and its positional parameters were refined freely [C—H = 0.87 (3)–1.05 (3) Å. All H atoms were refined with isotropic displacement parameters (*U*_iso_(H) = 1.2 *U*_eq_(CH) and 1.5*U*_eq_(CH_3_))

To improve the values of R1, wR2, and GOOF, the bad three reflections (1 1 0 2 0 0 1 1 1) were omitted from the refinement.

### Crystal data

**Table:**

C_15_H_14_O_2_	*F*_000_ = 480
*M**_r_* = 226.26	*D*_x_ = 1.196 Mg m^−^^3^
Monoclinic, *P*2_1_/*c*	Mo *K*α radiation λ = 0.71073 Å
Hall symbol: -P 2ybc	Cell parameters from 1609 reflections
*a* = 11.690 (2) Å	θ = 2.8–27.9º
*b* = 9.670 (1) Å	µ = 0.08 mm^−^^1^
*c* = 11.478 (2) Å	*T* = 299 (2) K
β = 104.50 (2)º	Prism, colourless
*V* = 1256.2 (3) Å^3^	0.50 × 0.46 × 0.20 mm
*Z* = 4	

### Data collection

**Table:**

Oxford Diffraction Xcalibur diffractometer with a Sapphire CCD detector	2529 independent reflections
Radiation source: fine-focus sealed tube	1385 reflections with *I* > 2σ(*I*)
Monochromator: graphite	*R*_int_ = 0.021
*T* = 299(2) K	θ_max_ = 26.4º
Rotation method data acquisition using ω and φ scans	θ_min_ = 2.8º
Absorption correction: multi-scan(CrysAlis RED; Oxford Diffraction, 2007)	*h* = −11→14
*T*_min_ = 0.968, *T*_max_ = 0.989	*k* = −12→9
7861 measured reflections	*l* = −13→14

### Refinement

**Table:**

Refinement on *F*^2^	Hydrogen site location: inferred from neighbouring sites
Least-squares matrix: full	H atoms treated by a mixture of independent and constrained refinement
*R*[*F*^2^ > 2σ(*F*^2^)] = 0.053	*w* = 1/[σ^2^(*F*_o_^2^) + (0.092*P*)^2^ + 0.342*P*] where *P* = (*F*_o_^2^ + 2*F*_c_^2^)/3
*wR*(*F*^2^) = 0.200	(Δ/σ)_max_ = 0.006
*S* = 1.04	Δρ_max_ = 0.21 e Å^−^^3^
2529 reflections	Δρ_min_ = −0.15 e Å^−^^3^
181 parameters	Extinction correction: SHELXL97 (Sheldrick, 2008), Fc^*^=kFc[1+0.001xFc^2^λ^3^/sin(2θ)]^-1/4^
Primary atom site location: structure-invariant direct methods	Extinction coefficient: 0.031 (6)
Secondary atom site location: difference Fourier map	

### Special details

**Table:**

Experimental. empirical absorption correction using spherical harmonics, implemented in SCALE3 ABSPACK scaling algorithm.
Geometry. All e.s.d.'s (except the e.s.d. in the dihedral angle between two l.s. planes) are estimated using the full covariance matrix. The cell e.s.d.'s are taken into account individually in the estimation of e.s.d.'s in distances, angles and torsion angles; correlations between e.s.d.'s in cell parameters are only used when they are defined by crystal symmetry. An approximate (isotropic) treatment of cell e.s.d.'s is used for estimating e.s.d.'s involving l.s. planes.
Refinement. Refinement of *F*^2^ against ALL reflections. The weighted *R*-factor *wR* and goodness of fit *S* are based on *F*^2^, conventional *R*-factors *R* are based on *F*, with *F* set to zero for negative *F*^2^. The threshold expression of *F*^2^ > σ(*F*^2^) is used only for calculating *R*-factors(gt) *etc*. and is not relevant to the choice of reflections for refinement. *R*-factors based on *F*^2^ are statistically about twice as large as those based on *F*, and *R*- factors based on ALL data will be even larger.

### Fractional atomic coordinates and isotropic or equivalent isotropic displacement parameters (Å^2^)

**Table:**

	*x*	*y*	*z*	*U*_iso_*/*U*_eq_	
C1	0.5348 (2)	0.2164 (2)	0.4626 (2)	0.0639 (6)	
C2	0.5493 (2)	0.1082 (2)	0.3892 (2)	0.0665 (6)	
C3	0.4583 (3)	0.0849 (3)	0.2881 (2)	0.0818 (8)	
H3	0.465 (2)	0.002 (3)	0.238 (3)	0.098*	
C4	0.3581 (3)	0.1645 (4)	0.2630 (3)	0.0920 (9)	
H4	0.293 (3)	0.134 (3)	0.192 (3)	0.110*	
C5	0.3464 (3)	0.2706 (4)	0.3381 (3)	0.0929 (10)	
H5	0.282 (3)	0.329 (3)	0.318 (3)	0.111*	
C6	0.4355 (3)	0.2979 (3)	0.4402 (3)	0.0787 (8)	
H6	0.436 (3)	0.363 (3)	0.493 (3)	0.094*	
C7	0.7074 (2)	0.3305 (2)	0.5779 (2)	0.0642 (6)	
C8	0.8038 (2)	0.3150 (2)	0.6887 (2)	0.0623 (6)	
C9	0.8060 (2)	0.2066 (3)	0.7680 (2)	0.0713 (7)	
H9	0.741 (2)	0.137 (3)	0.754 (2)	0.086*	
C10	0.8988 (2)	0.1943 (3)	0.8698 (2)	0.0774 (7)	
H10	0.901 (2)	0.116 (3)	0.926 (2)	0.093*	
C11	0.9911 (2)	0.2878 (3)	0.8946 (2)	0.0786 (7)	
C12	0.9879 (3)	0.3953 (3)	0.8146 (3)	0.0899 (9)	
H12	1.052 (3)	0.462 (3)	0.823 (3)	0.108*	
C13	0.8960 (2)	0.4098 (3)	0.7127 (3)	0.0802 (7)	
H13	0.896 (2)	0.489 (3)	0.650 (2)	0.096*	
C14	0.6588 (2)	0.0208 (3)	0.4190 (3)	0.0866 (8)	
H14A	0.6673	−0.0210	0.4965	0.129*	
H14B	0.7264	0.0777	0.4203	0.129*	
H14C	0.6527	−0.0500	0.3592	0.129*	
C15	1.0906 (3)	0.2722 (4)	1.0064 (3)	0.1079 (11)	
H15A	1.1586	0.2344	0.9851	0.162*	
H15B	1.0665	0.2111	1.0618	0.162*	
H15C	1.1101	0.3610	1.0434	0.162*	
O1	0.62322 (15)	0.23295 (16)	0.57090 (14)	0.0759 (5)	
O2	0.70209 (16)	0.41780 (18)	0.50210 (17)	0.0858 (6)	

### Atomic displacement parameters (Å^2^)

**Table:**

	*U*^11^	*U*^22^	*U*^33^	*U*^12^	*U*^13^	*U*^23^
C1	0.0637 (14)	0.0663 (13)	0.0593 (13)	−0.0023 (11)	0.0110 (11)	0.0084 (10)
C2	0.0723 (15)	0.0639 (13)	0.0640 (14)	0.0001 (11)	0.0183 (12)	0.0075 (11)
C3	0.097 (2)	0.0791 (17)	0.0654 (16)	−0.0007 (15)	0.0136 (14)	0.0045 (13)
C4	0.089 (2)	0.102 (2)	0.0721 (17)	−0.0112 (18)	−0.0030 (15)	0.0184 (17)
C5	0.0727 (19)	0.101 (2)	0.100 (2)	0.0197 (16)	0.0132 (17)	0.0367 (19)
C6	0.0850 (19)	0.0726 (15)	0.0808 (18)	0.0127 (14)	0.0252 (15)	0.0111 (13)
C7	0.0685 (15)	0.0544 (12)	0.0733 (15)	0.0050 (11)	0.0248 (12)	−0.0033 (11)
C8	0.0629 (13)	0.0598 (12)	0.0673 (14)	−0.0005 (10)	0.0220 (11)	−0.0064 (10)
C9	0.0678 (15)	0.0668 (14)	0.0770 (16)	−0.0082 (12)	0.0141 (13)	−0.0005 (12)
C10	0.0683 (16)	0.0829 (17)	0.0773 (17)	−0.0054 (13)	0.0115 (13)	0.0052 (13)
C11	0.0633 (15)	0.0930 (18)	0.0776 (17)	−0.0054 (13)	0.0140 (12)	−0.0117 (14)
C12	0.0740 (18)	0.0924 (19)	0.101 (2)	−0.0253 (15)	0.0165 (16)	−0.0105 (17)
C13	0.0813 (18)	0.0707 (15)	0.0899 (19)	−0.0129 (13)	0.0242 (15)	0.0009 (13)
C14	0.0866 (19)	0.0814 (17)	0.0954 (19)	0.0142 (14)	0.0294 (15)	0.0050 (14)
C15	0.0766 (19)	0.138 (3)	0.098 (2)	−0.0173 (18)	0.0013 (16)	−0.0083 (19)
O1	0.0790 (11)	0.0763 (11)	0.0667 (11)	−0.0141 (9)	0.0075 (8)	0.0060 (8)
O2	0.0901 (13)	0.0725 (11)	0.0936 (14)	0.0013 (9)	0.0205 (10)	0.0188 (9)

### Geometric parameters (Å, °)

**Table:**

C1—C6	1.373 (3)	C8—C13	1.390 (3)
C1—C2	1.381 (3)	C9—C10	1.386 (4)
C1—O1	1.413 (3)	C9—H9	1.00 (3)
C2—C3	1.383 (4)	C10—C11	1.382 (4)
C2—C14	1.499 (3)	C10—H10	0.99 (3)
C3—C4	1.371 (4)	C11—C12	1.382 (4)
C3—H3	1.00 (3)	C11—C15	1.508 (4)
C4—C5	1.369 (4)	C12—C13	1.383 (4)
C4—H4	1.01 (3)	C12—H12	0.97 (3)
C5—C6	1.384 (4)	C13—H13	1.05 (3)
C5—H5	0.92 (3)	C14—H14A	0.9600
C6—H6	0.87 (3)	C14—H14B	0.9600
C7—O2	1.202 (3)	C14—H14C	0.9600
C7—O1	1.351 (3)	C15—H15A	0.9600
C7—C8	1.481 (3)	C15—H15B	0.9600
C8—C9	1.385 (3)	C15—H15C	0.9600
			
C6—C1—C2	123.3 (2)	C10—C9—H9	118.6 (15)
C6—C1—O1	119.9 (2)	C11—C10—C9	121.6 (3)
C2—C1—O1	116.6 (2)	C11—C10—H10	118.4 (16)
C1—C2—C3	116.7 (2)	C9—C10—H10	120.0 (16)
C1—C2—C14	121.1 (2)	C12—C11—C10	117.7 (3)
C3—C2—C14	122.2 (2)	C12—C11—C15	121.9 (3)
C4—C3—C2	121.5 (3)	C10—C11—C15	120.4 (3)
C4—C3—H3	121.1 (16)	C11—C12—C13	121.7 (3)
C2—C3—H3	117.2 (16)	C11—C12—H12	122.7 (18)
C5—C4—C3	120.2 (3)	C13—C12—H12	115.5 (18)
C5—C4—H4	123.4 (18)	C12—C13—C8	120.0 (3)
C3—C4—H4	116.1 (18)	C12—C13—H13	121.3 (15)
C4—C5—C6	120.2 (3)	C8—C13—H13	118.6 (15)
C4—C5—H5	120.5 (19)	C2—C14—H14A	109.5
C6—C5—H5	119 (2)	C2—C14—H14B	109.5
C1—C6—C5	118.1 (3)	H14A—C14—H14B	109.5
C1—C6—H6	115.4 (19)	C2—C14—H14C	109.5
C5—C6—H6	126.5 (19)	H14A—C14—H14C	109.5
O2—C7—O1	122.9 (2)	H14B—C14—H14C	109.5
O2—C7—C8	125.6 (2)	C11—C15—H15A	109.5
O1—C7—C8	111.49 (19)	C11—C15—H15B	109.5
C9—C8—C13	118.9 (2)	H15A—C15—H15B	109.5
C9—C8—C7	121.8 (2)	C11—C15—H15C	109.5
C13—C8—C7	119.3 (2)	H15A—C15—H15C	109.5
C8—C9—C10	120.1 (2)	H15B—C15—H15C	109.5
C8—C9—H9	121.4 (15)	C7—O1—C1	119.55 (17)
			
C6—C1—C2—C3	0.9 (4)	C13—C8—C9—C10	0.4 (4)
O1—C1—C2—C3	174.9 (2)	C7—C8—C9—C10	178.9 (2)
C6—C1—C2—C14	−179.0 (2)	C8—C9—C10—C11	−0.4 (4)
O1—C1—C2—C14	−4.9 (3)	C9—C10—C11—C12	0.3 (4)
C1—C2—C3—C4	−0.7 (4)	C9—C10—C11—C15	179.7 (3)
C14—C2—C3—C4	179.1 (2)	C10—C11—C12—C13	−0.1 (4)
C2—C3—C4—C5	0.4 (4)	C15—C11—C12—C13	−179.5 (3)
C3—C4—C5—C6	−0.3 (5)	C11—C12—C13—C8	0.1 (4)
C2—C1—C6—C5	−0.7 (4)	C9—C8—C13—C12	−0.2 (4)
O1—C1—C6—C5	−174.6 (2)	C7—C8—C13—C12	−178.8 (2)
C4—C5—C6—C1	0.4 (4)	O2—C7—O1—C1	10.7 (3)
O2—C7—C8—C9	−176.4 (2)	C8—C7—O1—C1	−169.31 (18)
O1—C7—C8—C9	3.7 (3)	C6—C1—O1—C7	−85.0 (3)
O2—C7—C8—C13	2.2 (4)	C2—C1—O1—C7	100.8 (2)
O1—C7—C8—C13	−177.7 (2)		
